# Gradients of connectivity distance are anchored in primary cortex

**DOI:** 10.1007/s00429-016-1333-7

**Published:** 2016-11-02

**Authors:** Sabine Oligschläger, Julia M. Huntenburg, Johannes Golchert, Mark E. Lauckner, Tyler Bonnen, Daniel S. Margulies

**Affiliations:** 1 Max Planck Research Group for Neuroanatomy & Connectivity, Max Planck Institute for Human Cognitive and Brain Sciences, Stephanstraße 1a, 04317 Leipzig, Germany; 2 Faculty of Biosciences, Pharmacy and Psychology, University Leipzig, Leipzig, Germany; 3 Neurocomputation and Neuroimaging Unit, Department of Education and Psychology, Free University of Berlin, Berlin, Germany; 4 Department of Brain and Cognitive Sciences, Massachusetts Institute of Technology, Cambridge, MA USA

**Keywords:** Topography, Spatial organization, Connectivity, Cortical organization

## Abstract

Connectivity between distant cortical areas is a valuable, yet costly feature of cortical organization and is predominantly found between regions of heteromodal association cortex. The recently proposed ‘tethering hypothesis’ describes the emergence of long-distance connections in association cortex as a function of their spatial separation from primary cortical regions. Here, we investigate this possibility by characterizing the distance between functionally connected areas along the cortical surface. We found a systematic relationship between an area’s characteristic connectivity distance and its distance from primary cortical areas. Specifically, the further a region is located from primary sensorimotor regions, the more distant are its functional connections with other areas of the cortex. The measure of connectivity distance also captured major functional subdivisions of the cerebral cortex: unimodal, attention, and higher-order association regions. Our findings provide evidence for the anchoring role of primary cortical regions in establishing the spatial distribution of cortical properties that are related to functional specialization and differentiation.

**Electronic supplementary material:**

The online version of this article (doi:10.1007/s00429-016-1333-7) contains supplementary material, which is available to authorized users.

## Introduction

The distance between cortical regions is a major determinant of connectivity in the cortex (Ercsey-Ravasz et al. 2013; Roberts et al. 2016; Betzel et al. 2016). Most cortico-cortical connections project locally (Markov et al. 2011), making the occurrence of long-distance connections a noteworthy feature of cortical organization. Perspectives from graph theory propose that distant connections balance their higher wiring cost by improving network efficiency (Kaiser and Hilgetag 2006; Bassett and Bullmore 2006; Achard and Bullmore 2007; Bullmore and Sporns 2012; Collin et al. 2014; Hahn et al. 2014). This concept of network optimization can account for the observed frequencies of connection lengths. However, distant projections are not evenly dispersed throughout the brain, rather demonstrating higher density between regions of heteromodal association cortex (Mesulam 1990; Sepulcre et al. 2010). Understanding their uneven spatial distribution requires additional principles unique to the development and functional organization of the cerebral cortex.

In contrast to the long connections extending between heteromodal association areas, connections within unimodal cortex—especially within primary sensory and motor areas—are largely restricted to adjacent areas (Felleman and Van Essen 1991; Sepulcre et al. 2010). The distance between interconnected areas can thus be taken as a distinguishing feature between unimodal sensorimotor cortex and the large-scale networks of heteromodal association regions (Sepulcre et al. 2010; Buckner and Krienen 2013).

Theories of cortical differentiation emphasize primary cortical regions for establishing the spatial layout of the cortex: locations of primary cortex are proposed to serve as anchor points around which other areas are arranged (Rosa and Tweedale 2005; Buckner and Krienen 2013). Buckner and Krienen (2013) further articulated this theory as the ‘tethering hypothesis’. They propose that the unique characteristics of association regions—such as their long-range reciprocal connections—emerge as a result of their distance from developmental constraints and hierarchical network structure that determine the specialization of primary regions during cortical ontogenesis. Untethered from these constraints, association cortex gains its specialized functions through the long-distance connections it forms.

Here, we investigate the spatial distribution of distances between interconnected areas within the framework of the tethering hypothesis. We hypothesize that the further a region is situated from primary cortex, the more distant are its connections. To address these questions, we (1) describe each point on the cortical surface based on its average geodesic distance to highly connected areas (which we will refer to as ‘distance-to-connected-areas’), (2) investigate its spatial covariance with respect to the locations of primary cortex, and (3) explore the relationship with functional networks. Unlike Euclidean distance, which has been previously implemented as a heuristic of white matter paths, geodesic distance measures distance along the cortical sheet and thus enables us to directly assess the implications of relative spatial positions across the cortical surface. In the current study, the geodesic distance between two points on the cortical surface refers to the shortest direct path along the surface mesh. Unlike the geodesic distance measure used in graph theory, which measures the number of edges between points, the current implementation measures spatial distance.

## Materials and methods

### Data acquisition

#### Participants

Data were obtained as part of a larger project conducted at the Max Planck Institute for Human Cognitive and Brain Sciences, Leipzig, Germany. Recruitment criteria were as follows: no previous or current clinical diagnosis of psychiatric or neurological condition, no drug consumption in the last six months, and MRI compatibility. For the current analysis, age was restricted to a range between 18 and 40 years to mitigate developmental and aging-related variance in brain structure and function. Insufficient quality of imaging data was determined by excessive head motion (mean framewise displacement >2 mm), poor coregistration according to visual inspection, and poor signal-to-noise ratio. These were considered exclusion criteria for the current analysis. The final sample consisted of *N* = 77 subjects (41 female, mean age 25.3 years, stand. dev. 3.6 years). All subjects gave written informed consent. The study was approved by the Ethics Committee of the Faculty of Medicine, Leipzig University.

#### MRI data

Magnetic resonance imaging (MRI) data were collected on a Siemens Magnetom Verio 3 Tesla scanner. A structural image was acquired using an MP2RAGE sequence (TR = 5000 ms, TE = 2.92 ms, TI1 = 700 ms, TI2 = 2500 ms, flip angle 1 = 4°, flip angle 2 = 5°, voxel size = 1.0 mm isotropic, duration = 8.22 min; Marques et al. 2010). Four resting-state functional MRI scans were acquired using a multiband EPI sequence (TR = 1400 ms, TE = 39.4 ms, flip angle = 69°, multiband acceleration factor = 4, voxel size = 2.3 mm isotropic, 64 slices, 657 volumes, duration = 15.30 min; Feinberg et al. 2010; Moeller et al. 2010). Sequences were identical across runs with the exception of alternating phase encoding and slice orientation (AC–PC axis and aligned to orbitofrontal cortex) to vary the spatial distribution of signal loss across runs. During resting-state scans, participants were instructed to fixate on a crosshair. Field maps (TR = 680 ms, TE1 = 5.19 ms, TE2 = 7.65 ms, flip angle = 60°, voxel size = 2.3 mm isotropic, 64 slices) were obtained separately for each resting-state run to correct for magnetic field inhomogeneities.

### Data analysis

#### MRI preprocessing

MRI data were preprocessed using FSL 5.0, FreeSurfer 5.3.0, AFNI, ANTs 2.1.0-rc3, dcmstack 0.7.dev, C3D 1.0.0, CBS Tools 3.0 and streamlined in a reusable pipeline^1^ using Nipype (Gorgolewski et al. 2011).

For structural preprocessing, the background of each subject’s T1-weighted image was removed using CBS Tools (Bazin et al. 2014). The masked image was used for cortical surface reconstruction using FreeSurfer’s full version of recon-all (Dale et al. 1999; Fischl et al. 1999).

For functional preprocessing, the first five volumes of each resting-state run were excluded. Spatial transformations of the functional volumes included motion correction, distortion correction, and coregistration to the structural image. Transformation parameters for motion correction were obtained by rigid-body realignment to the sixth volume of the original time series (Jenkinson et al. 2002). A temporal mean image of the realigned time series was rigidly registered to the field map magnitude image (Jenkinson and Smith 2001) and unwarped (Jenkinson et al. 2012) to estimate transformation parameters for distortion correction. The unwarped temporal mean was then rigidly coregistered to the subject’s structural scan (Greve and Fischl 2009) yielding transformation parameters for coregistration. The obtained spatial transformations were then combined and applied to each volume of the original time series in one single interpolation. To mitigate remaining motion artifacts, the six motion parameters and their first derivatives were included as nuisance regressors in a general linear model (GLM). They were regressed out for each voxel’s time series along with regressors representing intensity-defined temporal outliers (Nipype’s rapidart^2^), and linear and quadratic trends. From the residual time series, six principal components of signal fluctuations in white matter and cerebrospinal fluid (assumed to reflect physiological noise) were derived and included as additional regressors in a second GLM (Behzadi et al. 2007). The denoised time series were temporally filtered to a frequency range between 0.01 and 0.1 Hz, mean centered and variance normalized (Rokem et al. 2008).

The preprocessed time series in each subject’s native volume space were then sampled to the fsaverage5 surface template (FreeSurfer mri_vol2surf). First, for each node on the subject’s native surface mesh, time series were sampled from voxels within the central 80% cortical depth along the surface normal, then averaged and projected to the surface. Time series were mapped and down-sampled to the template surface using spherical surface registration and were spatially smoothed with a Gaussian kernel of 6 mm FWHM.

In 15 subjects, slight imprecision in coregistration affected the most posterior nodes of the occipital pole. In such cases, the surface outline of the anatomical image traced gray matter beyond the extent of the functional image, leaving some surface nodes with no data to sample from during surface projection. On average, eight nodes were affected in this subgroup (0.07% of the total number of nodes), with the highest number being 145 (1.4%). For these nodes, time series were spatially interpolated on the surface by iteratively averaging from immediate neighbors that contain functional data. The interpolation process inflates similarity between neighboring regions—thereby, artificially increasing their local connectivity and distorting the measure of distance-to-connected-areas presented here. Masking out the affected regions at the subject level would have led to varying numbers of nodes across subjects, which is problematic for group averaging. On the other hand, creating a mask at the group level would have led to complete data loss for the affected nodes, despite the majority of subjects having good data there. Hence, we opted for spatial interpolation, having its shortcomings and implications for the proposed measure in mind.

#### Subject-level analysis^3^

##### Functional connectivity

For each subject, connectivity matrices were created separately for each hemisphere. Functional connectivity between each pair of cortical nodes was quantified by temporal correlation of their time series (Pearson product–moment correlation coefficient) and entered in a node-by-node matrix of functional connectivity. Correlation matrices were created for each resting-state scan separately and combined across scans by averaging matrices.

##### Distance along the cortical surface

Distance along the cortical surface was measured by an algorithm for approximating the exact geodesic distance on triangular meshes (O’Rourke 1999).^4^ In contrast to a straight line in three-dimensional space (Euclidean distance), geodesic distance describes the shortest path between two nodes along the cortical surface and captures the relative spatial layout of regions within the cortical surface. Distances were quantified on each subject’s native surface mesh. Correspondence with template space was achieved via spherical surface meshes. Each fsaverage5 node was assigned to the closest node within the native space. This resulted in a node-by-node matrix of geodesic distance for each hemisphere of the subject in fsaverage5 space. To adjust for differences in brain size, the overall distance distribution for each subject was normalized to a range between 0 and 1. For comparison, we repeated this analysis using Euclidean distance.

##### Characterizing distance-to-connected-areas

**Fig. 1:**
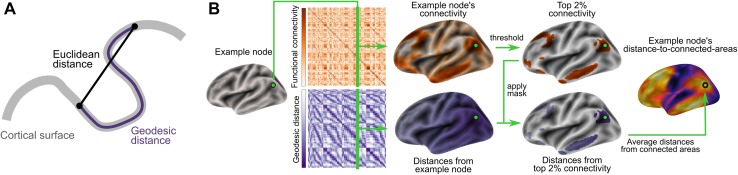
Calculation of distance-to-connected-areas. **a** Geodesic distance. To capture spatial relationships of areas across the cortical surface, we measured the geodesic distance between nodes. In contrast to Euclidean distance (i.e., a straight line between two points; *black*), geodesic distance refers to the shortest path along the surface (*purple*). **b** Maps of distance-to-connected-areas describe each node’s average distance to functionally connected areas along the cortical surface. Computation for one example node: the ipsilateral functional connectivity for this node was thresholded to select nodes of highest connectivity only. The seed node’s geodesic distance to all selected nodes was averaged resulting in its characteristic distance-to-connected-areas

We computed each node’s distance-to-connected-areas by its average geodesic distance to functionally connected nodes. The workflow is illustrated in Fig. 1. For each node separately, connectivity was thresholded by the node’s 2% highest connectivity. Distances to these nodes were then averaged, yielding the overall distance-to-connected-areas for that node. To avoid results specific to an arbitrary threshold choice, the workflow was repeated for a range of percentiles (top 30, 25, 20, 15, 10, 5%). Applying a node-wise threshold instead of defining an overall threshold attempts to adjust for differences in correlation strengths across nodes. Maps of distance-to-connected-areas were created for each resting-state scan separately as well as for the combined ones. For comparison, they were also calculated using Euclidean distances.

#### Group-level analysis^5^

##### Group maps of distance-to-connected-areas

For group-level maps of distance-to-connected-areas, the individual maps based on the combined scans were averaged across subjects. For visualization, values across the surface were projected onto the inflated cortical surface.^6^ For ease of interpretation, the distance was rescaled to millimeters.

##### Spatial distribution of distance-to-connected-areas

To investigate the anchoring role of primary cortex in the spatial distribution of connectivity distance, group-level distance-to-connected-areas was correlated with distance from primary cortex. A map of distance to the nearest primary cortex was created as follows: fsaverage5 FreeSurfer labels of the calcarine sulcus, temporal transverse sulcus, and central sulcus served to demarcate the cortical landmarks of primary cortex. Seed regions for calculating geodesic distance were taken from the sulcal depths of the labels (outlined in green, Online Resource Fig. 1). Geodesic distance to the closest seed node was assigned to each node outside these seed regions. In a secondary step, the prefrontal areas anterior to the intermediate frontal sulcus were excluded from the correlation as they deviated from the overall pattern of increasing connectivity distance with distance from primary cortex (Fig. 3a).

We then assessed how frequent the arrangement of distance-to-connected-areas and its systematic relationship with distance from primary cortex might occur by chance. To this end, network topography was permuted and both the distance-to-connected-areas and its relationship to the new locations of primary cortex were recalculated. Specifically, retaining the number of spatially continuous patches in the 17-network parcellation (*n* = 42 comprising network patches >25 vertices; Yeo et al. 2011), we generated random patches on the cortical surface and matched them by size to the original network patches. Functional connectivity in this new network arrangement was modeled using a binary graph with edges linking the surface vertices within, but not between networks. For each new network topography and its corresponding model connectivity, the distance-to-connected-areas and its topographical correlation with new locations of primary cortex was computed. This procedure was iterated 1000 times to generate an empirical frequency distribution of correlations under random network topography. This procedure was repeated using the original network arrangement and compared to the frequency distribution based on random topography to estimate the probability of a systematic relationship between distance-to-connected-areas and distance from primary cortex under a random network topography.

##### Distance-to-connected-areas and functional networks

For each network (17-network template by Yeo et al. 2011), the distance-to-connected-areas within its spatial extent was sampled to create network-specific distributions of underlying distance-to-connected-areas. Sorting distributions by increasing mean suggested three groups of networks. To confirm this observation, distributions were then compared between each pair of networks using the Jensen–Shannon divergence measure. *k*-means clustering was applied to the Jenson–Shennon divergence matrix to group the networks (Fig. 4c).

##### Post hoc tests on method choices

To avoid obtaining results specific to an arbitrary threshold choice, we assessed the effect of several thresholds (30, 25, 20, 15, 10, 5, 2% highest connectivity per node) on the resulting group-level maps, the standard deviation across the group, and their test–retest reliability (Online Resource Fig. 2a–c). Test–retest reliability was measured using the intraclass correlation coefficient (ICC). Furthermore, we assessed the effect of threshold on defining consistent groups of networks (as described above, Online Resource Fig. 3).

We compared maps of geodesic distance-to-connected-areas with those based on Euclidean distance. Subject-specific difference maps were created by subtracting Euclidean from geodesic maps. Difference maps were then averaged across subjects to identify regions of high discrepancy at the group level (Online Resource Fig. 2d).

## Results

### Distance-to-connected-areas increases with distance from the primary cortex

**Fig. 2:**
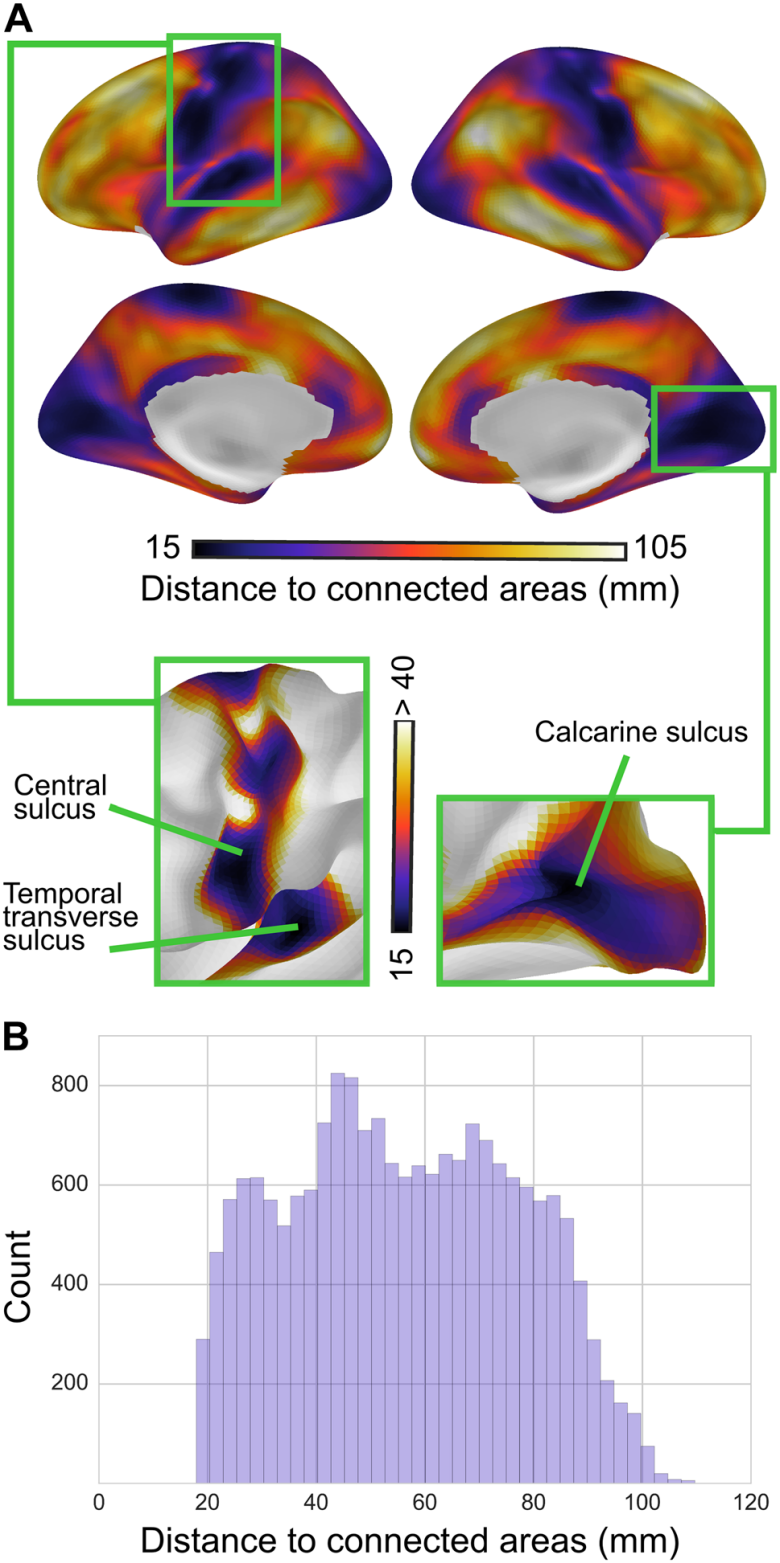
Group-level distance-to-connected-areas. **a** Group-level maps of distance-to-connected-areas (at 2% connectivity threshold) formed a consistent topographical pattern: distance-to-connected-areas was shortest in primary cortex and longest in association cortex. Specifically, the measure precisely delineated primary cortical regions: shortest distances lined the temporal transverse sulcus (primary auditory cortex, *left box*), the central sulcus (primary motor and somatosensory cortex, *left box*), and the calcarine sulcus (primary visual cortex, *right box*). With further distance from these regions, distance-to-connected-areas increased progressively, reaching peak values in higher-order association areas (lateral temporal, inferior parietal lobule, superior and middle frontal gyri). **b** *Histogram* of group-level distance-to-connected-areas

Distance-to-connected-areas was shortest in primary sensorimotor regions and peaked in higher-order association areas (Fig. 2). Notably, the shortest distances precisely delineated cortical landmarks of primary areas, bounding the lip of the calcarine sulcus that delineates the primary from the secondary visual cortex, the temporal transverse sulcus of the primary auditory cortex, and the upper banks of the central sulcus, which demarcates the primary motor and somatosensory cortex. At the 2% connectivity threshold, distance-to-connected-areas in all primary regions consistently ranged between 15 and 40 mm. With further distance from these regions, the distance-to-connected-areas progressively increased, peaking at approximately 100 mm in regions of the superior temporal sulcus, middle temporal gyrus, angular gyrus, precuneus, and caudal prefrontal cortex. One notable exception was located within the caudalmost portion of the intermediate frontal sulcus, homologous to cytoarchitectonically defined area 46, which demonstrated a decrease in distance-to-connected-areas compared to adjacent prefrontal regions. Other regions showing relatively short distance-to-connected-areas compared to surrounding regions included the posterior cingulate cortex (PCC), rostral anterior cingulate cortex (ACC), and posterior insula.

**Fig. 3:**
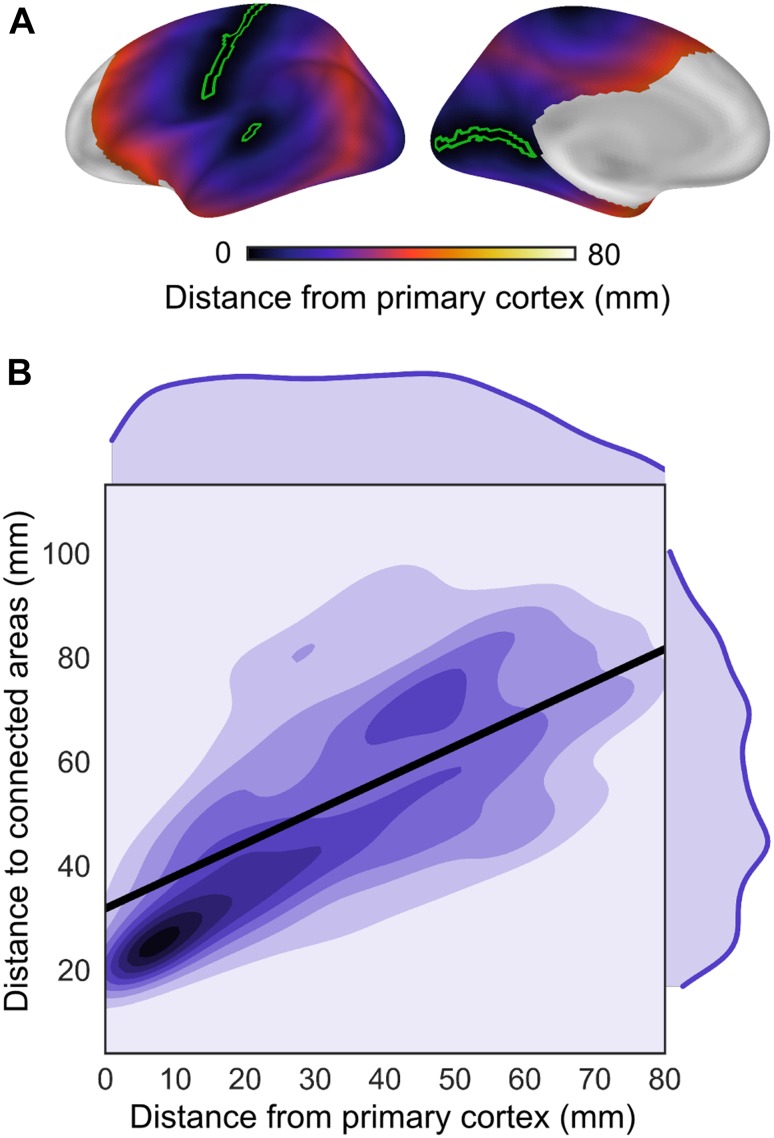
The spatial progression of distance-to-connected-areas across the cortical surface was investigated with relation to locations of primary cortex. **a** Distance from primary cortex. The map shows the geodesic distance from the closest node of a primary cortical region (*green outline*: depth of the calcarine sulcus, temporal transverse sulcus, and central sulcus). Prefrontal areas anterior to the intermediate frontal sulcus were excluded from the correlation as they deviated from the overall pattern of increasing connectivity distance with distance from primary cortex. **b** Distance-to-connected-areas showed a systematic relationship with distance from primary cortex as shown by the spatial correlation between the map of distance-to-connected-areas (shown in Fig. 2) and the map of distance from primary cortex (Spearman’s *r* = 0.7, *p* ≤ 0.01, slope = 0.6). These findings show that the spatial distribution of distance-to-connected-areas is anchored in locations of primary cortex

We investigated this spatial arrangement further by comparing the map of distance-to-connected-areas with that of geodesic distance from primary sensorimotor areas (Fig. 3a). In general, the further an area was located from the primary cortical landmarks, the greater were its distance-to-connected-areas (Spearman’s *r* = 0.7, *p* ≤ 0.01, slope = 0.6; Fig. 3). This relationship was present throughout the cortex, with the exception of prefrontal areas anterior to the intermediate frontal sulcus, described above.

To estimate how frequent this systematic relationship might occur by chance, we assessed the probability of correlation between the distance-to-connected-areas and the distance from primary cortex under random network topography. Correlations between connectivity distance and distance from primary cortex were distributed normally between *r* = −0.54 and *r* = +0.66. The binary connectivity model based on the original network arrangement captured the topography of connectivity distance well (spatial correlation of *r* = 0.74 between the original and model distance-to-connected-areas; Online Resource Fig. 4a). Here, the correlation between distance-to-connected-areas and distance from primary cortex was *r* = 0.59, corresponding to a probability of *p* < 0.002 to occur by chance under random network topography (Online Resource Fig. 4b).

### Distance-to-connected-areas distinguishes classes of functional networks

**Fig. 4:**
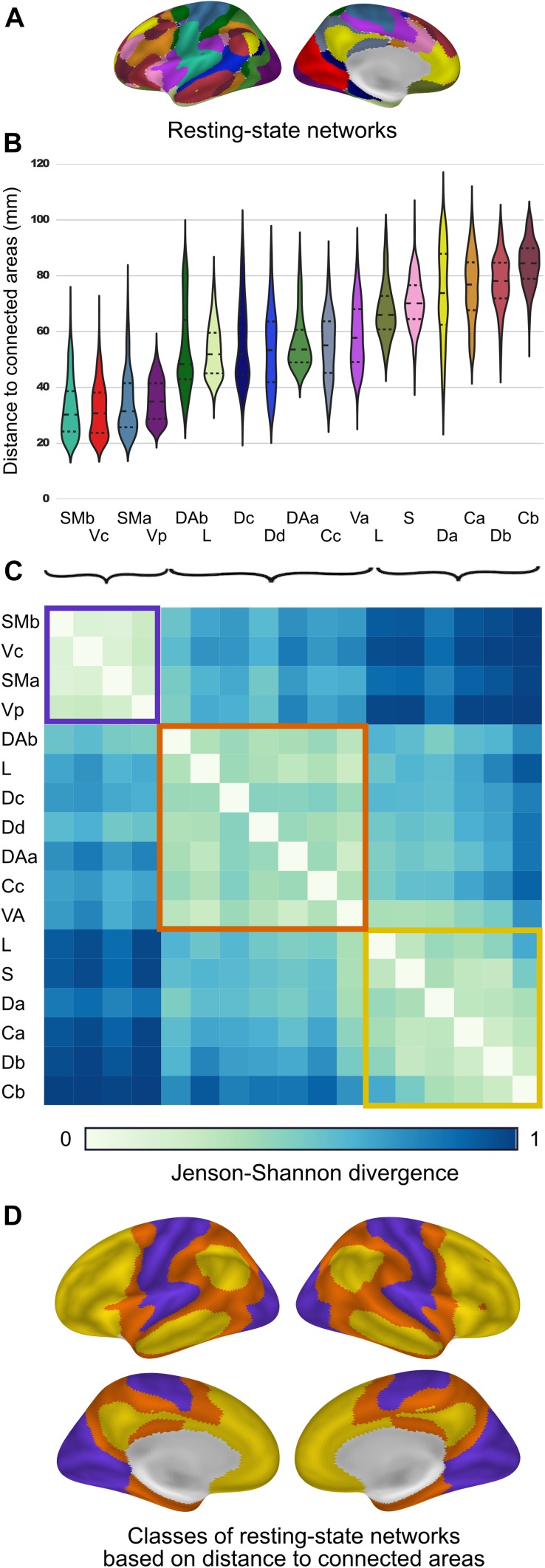
Broad domains of cortical functions are marked by distance-to-connected-areas. **a** Intrinsic networks (17-network template by Yeo et al. 2011). **b** Network-specific distributions of distance-to-connected-areas. **c** This matrix shows pairwise comparisons between the network distributions using the Jenson–Shannon divergence measure. *k*-means clustering of this matrix revealed three network groups that are broadly similar in functionality: sensorimotor (*purple*), attention (*orange*), and higher-order cognitive functions (*yellow*). **d** Class membership projected to the fsaverage5 surface. *SMb* somatomotor, *Vc* visual, *SMa* somatomotor, *Vp* visual, *DAb* dorsal attention, *L* limbic, *Dc* default, *Dd* default, *DAa* dorsal attention, *Cc* frontoparietal control, *VA* ventral attention, *S* salience, *Da* default, *Ca* frontoparietal control, *Db* default, *Cb* frontoparietal control

Intrinsic functional networks (Yeo et al. 2011; 17-network template in Fig. 4a) systematically differed in their distance-to-connected-areas. Figure 4b shows the distribution of distance-to-connected-areas specific to each network. Using Jenson–Shannon divergence as a distance metric between all pairs of distributions, *k*-means clustering was applied to identify three network groups (Fig. 4c, d). Reflecting the spatial continuity of sensory and motor networks, the distance-to-connected-areas was shortest in visual and somatomotor networks (purple cluster in Fig. 4c). In general, intermediate distance-to-connected-areas was observed in the dorsal and ventral attention networks (orange cluster in Fig. 4c), while the highest distances were present in the default mode and fronto-parietal control networks consistent with their distributed nature (yellow cluster in Fig. 4c).

### Impact of connectivity threshold and distance measure

While maps of distance-to-connected-areas largely corresponded across different thresholds (30–2% highest connectivity per node), there were some notable deviations associated with both more lenient as well as stricter threshold choices.

Distances decreased with stricter thresholds (Online Resource Fig. 2a), as would be expected given the inverse relationship between connection strength and length (Ercsey-Ravasz et al. 2013). At stricter thresholds (2–5% highest connectivity per node), maps showed greater spatial variance in distance-to-connected-areas (Online Resource Fig. 2a) and precisely delineated primary cortical regions (see Fig. 2). Lenient thresholds (20–30% highest connectivity per node) did not provide a clear delineation between the primary visual and adjacent association cortex (Online Resource Fig. 2a). Similarly, stricter thresholds resulted in more functionally coherent network clusters (Online Resource Fig. 3).

At lenient thresholds (20–30% highest connectivity per node) the visual cortex demonstrated among the highest distance-to-connected-areas despite being unimodal. We attribute this finding to the overall shape of the cortex. At these thresholds, a node’s average distance includes major parts of the cortex—and hence a region’s *position* within the cortical surface influences its distance-to-connected-areas.

Distance-to-connected-areas varied more across individuals using stricter thresholds (Online Resource Fig. 2b). For all thresholds, regions of greater distance-to-connected-areas tended to vary more across individuals (Online Resource Fig. 2b). ICC did not show a notable dependence on threshold choice (Online Resource Fig. 2c). ICC was lower in regions impacted by susceptibility artifacts such as orbitofrontal cortex.

Overall, maps of distance-to-connected-areas were similar using either geodesic or Euclidean distance—especially when using strict thresholds (Online Resource Fig. 2d). For all thresholds, areas of longer distance-to-connected-areas showed a greater difference between the two distance metrics. Geodesic distance-to-connected-areas appeared to capture more variance across the cortex than Euclidean distance (Online Resource Fig. 2a).

## Discussion

Our findings are consistent with previous maps of cortical connectivity distance (Sepulcre et al. 2010). The current study introduced the geodesic distance metric to measure distance along the cortical surface, preserving relative spatial positions irrespective of folding or expansion (Griffin 1994). We found that a region’s distance to functionally connected cortical areas increased with distance from primary sensorimotor regions. By describing the average distance-to-connected-areas from each point on the cortical surface, we observed a consistent topographic pattern of shortest distances within unimodal cortex and longest within heteromodal cortex (Fig. 2). Further analysis of this spatial progression revealed a more subtle relationship with the location of primary cortical landmarks (Fig. 3). Rather than a sharp distinction between uni- and heteromodal regions, we observed a linear increase in connectivity distance the further regions were located from the primary cortex. This relationship was attributable to precise topographical organization of connectivity in the cortex, as it was found unlikely to occur under random network topography (*p* < 0.002). These findings point to the anchoring role of primary cortical regions in establishing the spatial organization of connectivity patterns.

The role of primary areas as cornerstones for the spatial arrangement of surrounding regions finds support in theories from both cortical ontogeny and phylogeny. According to the tethering hypothesis (Buckner and Krienen 2013), association cortex emerges owing to the cortical expansion that buffers it from the constraints of molecular gradients and thalamic inputs that lead to the specialized differentiation of primary areas. The properties of association cortex are proposed to result from being untethered from the hierarchies imposed by primary regions. In the present study, the longest distance-to-connected-areas occurred in regions of disproportionate cortical expansion during primate evolution (Hill et al. 2010). Under this framework, we hypothesize consistent topographic patterns of distance-to-connected-areas in other primates, with differences in the proportion of cortex dedicated to distributed and distant connectivity.

Maps of cortical myelination (Glasser and Van Essen 2011) and cortical thickness (Wagstyl et al. 2015) follow similar spatial gradients and distinguish unimodal from heteromodal association cortex. Wagstyl et al. (2015) showed that cortical thickness increases with geodesic distance from primary regions. Their results also revealed a relationship with structural hierarchies in the macaque monkey, linking distance from primary cortex to cytoarchitectonic differentiation. Their findings provide further support for the anchoring role of primary cortex in shaping cortical differentiation.

We observed several deviations from the overall pattern of long distance-to-connected-areas for regions far from primary cortex. One such deviation was a marked decrease in connectivity distance in the caudalmost portion of the intermediate frontal sulcus, homologous to cytoarchitectonically defined area 46. This decrease in connectivity distance compared to surrounding regions is due to its extensive connectivity with premotor and inferior parietal cortex, adjacent to the pre- and postcentral sulcus (cf. ventral attention network as noted in Yeo et al. 2011). Other regions that departed from the overall pattern included rostral ACC, posterior insula, and PCC. In addition to primary sensorimotor regions, these regions, too, demonstrated relatively short connectivity distances. The rostral ACC and posterior insula, in particular, constitute interoceptive cortex, which is involved in the perception of the body’s internal state (Barrett and Simmons 2015). Observing a similar distribution of shorter connectivity distance in both interoceptive and exteroceptive cortex (e.g., visual, auditory, and somatosensory cortex) suggests that patterns of progressively longer distance-to-connected-areas may constitute an organizational feature underlying cortical processing hierarchies.

The distance between areas is a major determinant of cortical connectivity (Ercsey-Ravasz et al. 2013; Roberts et al. 2016; Betzel et al. 2016). However, while the overall relationship of connectivity with distance can account for various topological features, it cannot account for the location of hubs in association cortex (Roberts et al. 2016). The maps of distance-to-connected-areas presented here show that the relationship of connectivity with distance is not the same across cortical regions. The variance of this relationship—especially its progression of increasing connectivity distance toward the association cortex—and the spatial distribution of cortical hubs might be related to a common developmental mechanism. A recent model of brain network development explored possible mechanisms by which long-range connection form in the cortex. The model suggests that areas which develop earlier in network formation can establish longer connections (Kaiser et al. 2009). As a mature layer structure appears earlier within association areas during neuronal migration (Rakic 2002), it is reasonable that these areas begin forming their connections sooner, giving rise to the pattern of distance-to-connected-areas we observe.

Overall, we found shorter distance-to-connected-areas for stronger functional connectivity (Online Resource Fig. 2a: distance-to-connected-areas decreases with stricter connectivity thresholds). This pattern resembles findings from tract-tracing studies that quantified cortico-cortical connections in the macaque monkey. For example, Markov and colleagues (2011, 2013) found that local connections form the vast majority throughout the cortex with little discriminative power between regions, whereas long-range connections are sparse and low weight, but specific to a region’s connectivity profile. In this regard, the existence of only a few strong functional connections between distant areas may reflect the sparsity of long-range connections in the structural connectome. Our findings provide evidence that this sparsity systematically varies across the cortex and is mediated by proximity to the primary sensorimotor cortex. The higher occurrence of distant connectivity in regions of distributed networks (e.g., default mode and frontoparietal networks) further suggests that the similarity of connectivity profiles among these regions is less dependent on their mutual distance from each other (cf. Markov et al. 2013).

Approaches such as stepwise connectivity (Sepulcre et al. 2012) or network depth (Taylor et al. 2015) characterize cortical regions in terms of their *topological* distance from primary cortex (describing the number of connection steps traversed across the functional network graph) and have been interpreted in terms of perceptual convergence, information integration (Sepulcre et al. 2012; Sepulcre 2014), and functional abstraction (Taylor et al. 2015). Here, we assessed the *topographical* distance of regions from primary cortex (describing the geodesic distance along the cortical surface). The three network groups of distance-to-connected-areas (unimodal, attention and higher-order cognition) largely overlap with the three main stages of stepwise connectivity (primary/secondary, multimodal, cortical hubs). Progressive increases in distance-to-connected-areas may be one of the structural features by which the cortex achieves hierarchies of information integration and perceptual convergence.

In summary, we observe a trend of increasing distance to functionally connected areas that spatially progresses with distance from primary cortical areas. These findings indicate the importance of integrating the topography of cortical connectivity into the search for wiring principles and their relationship to functional specialization.

## Electronic supplementary material

Below is the link to the electronic supplementary material.

Supplementary material 1 (DOCX 4847 kb)

## Footnotes

1
https://github.com/NeuroanatomyAndConnectivity/pipelines/blob/master/src/lsd_lemon/lsd_resting.py.

2
http://nipy.org/nipype/interfaces/generated/nipype.algorithms.rapidart.html.

3
https://github.com/soligschlager/distconnect/tree/master/subject_level

4
https://code.google.com/archive/p/geodesic/ and https://github.com/margulies/topography/tree/master/utils/dist_matrix.

5
https://github.com/soligschlager/distconnect/tree/master/group_level

6
https://github.com/juhuntenburg/brainsurfacescripts/blob/master/plotting.py.
