# Supplementary material for: Gradients of connectivity distance are anchored in primary cortex
Source: Brain Struct Funct. 2016 Nov 2;222(5):2173–82. doi: 10.1007/s00429-016-1333-7 (PMC5504232; doi:10.1007/s00429-016-1333-7)
Supplement: Supplementary file 1 — Supplementary material 1 (DOCX 4847 kb) [file 429_2016_1333_MOESM1_ESM.docx]

# Online Resource

Brain Structure and Function

Gradients of connectivity distance are anchored in primary cortex

Sabine Oligschläger, Julia M. Huntenburg, Johannes Golchert, Mark E. Lauckner, Tyler Bonnen, Daniel S. Margulies^*^

* Correspondence:

Max Planck Institute for Human Cognitive and Brain Sciences

Stephanstraße 1a, 04317 Leipzig, Germany

+49 (0) 176 840 30 266

margulies@cbs.mpg.de

### Online Resource Figure 1


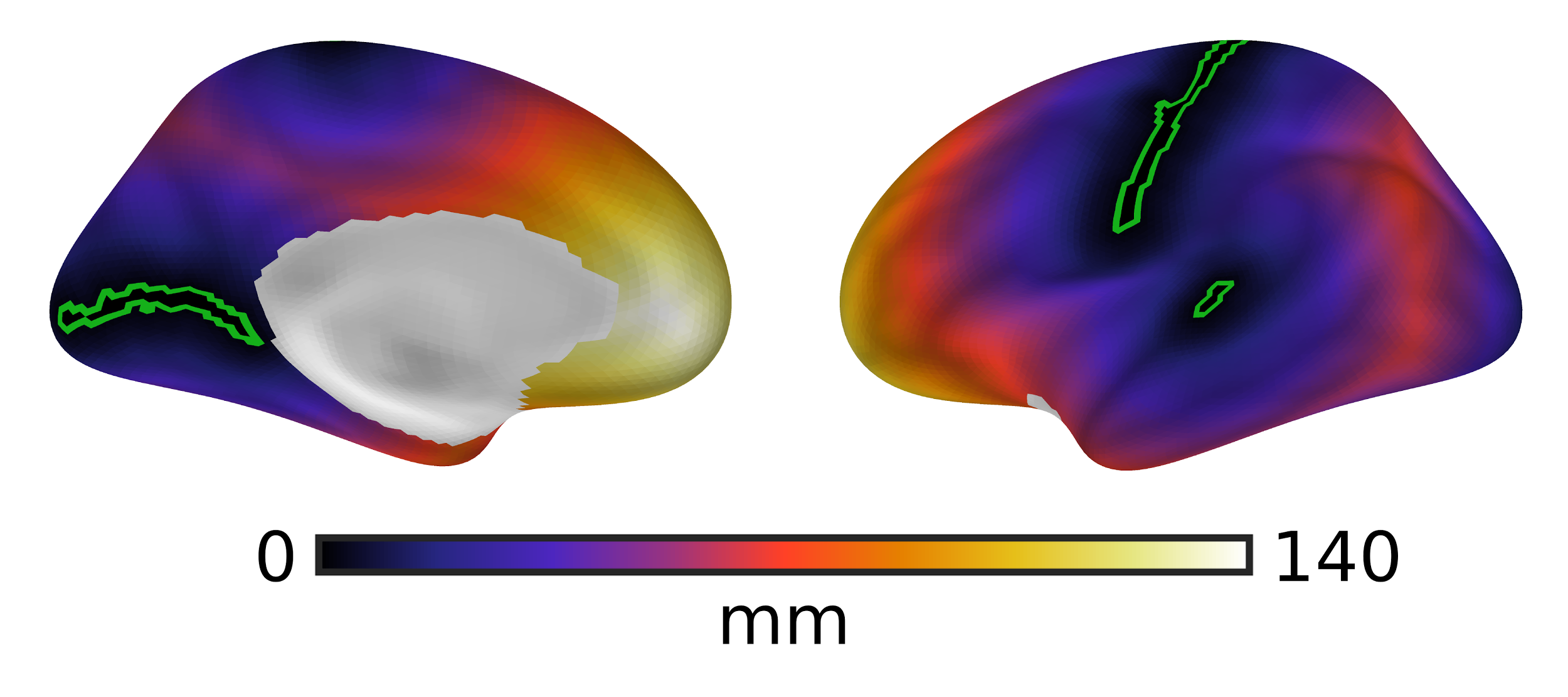


Distance from primary cortex. The map shows geodesic distance from the closest node of a primary cortical region (green outline: calcarine sulcus, temporal transverse sulcus, and central sulcus).

### Online Resource Figure 2


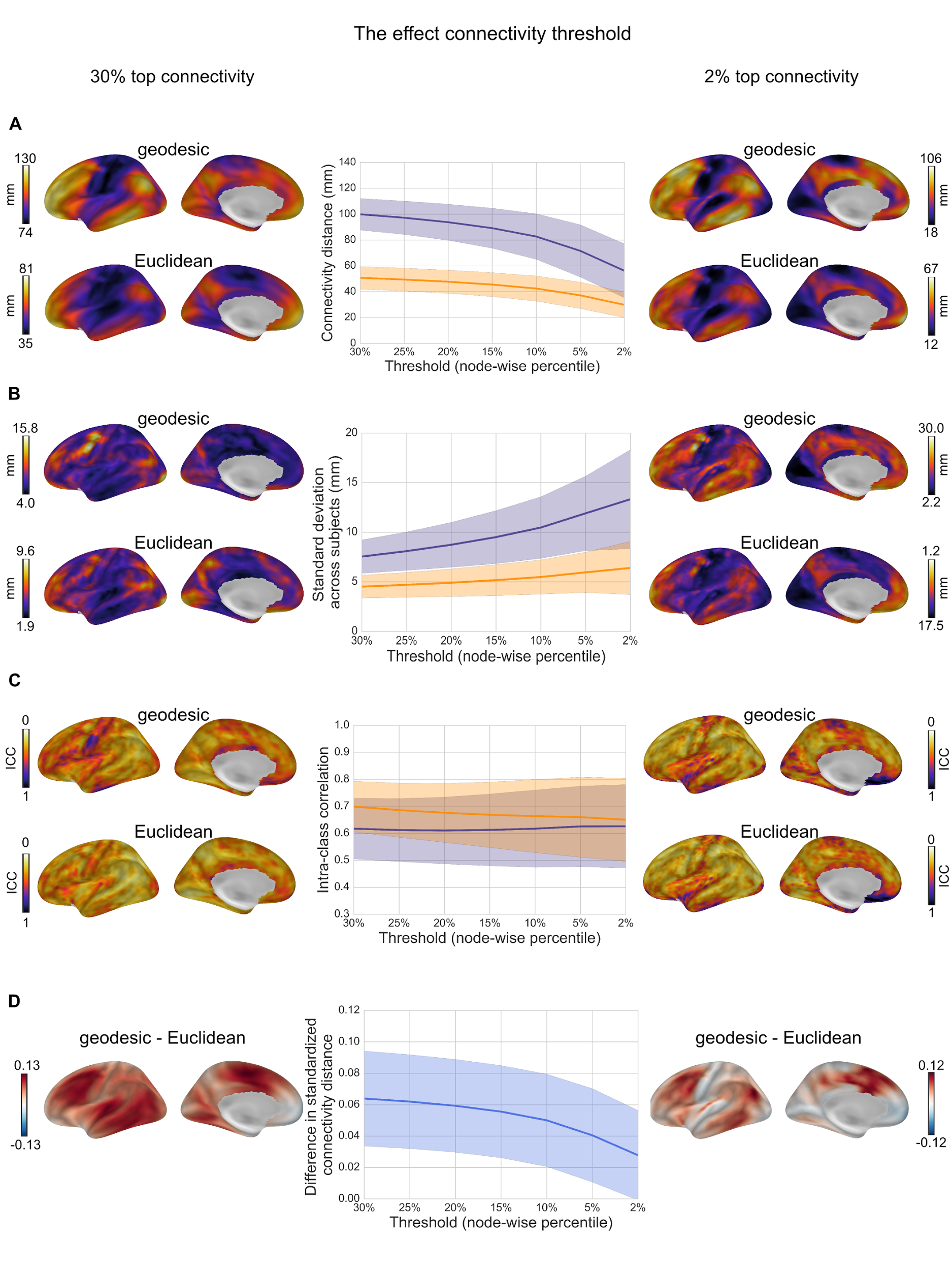


**Effects of connectivity threshold and distance metric on distance-to-connected-areas.** Different thresholds (30, 25, 20, 15, 10, 5, 2% highest connectivity per node) were explored. In (A), (B), and (C) lines refer to the mean and shades to the standard deviation of values within a map (purple for geodesic, orange for Euclidean distance-to-connected-areas). (A) Group-level distance-to- connected-areas as a function of threshold. Maps of distance-to-connected-areas were similar across thresholds. Overall, distance decreased with stricter thresholds. Stricter thresholds (e.g. 2-5 % connectivity per node) showed a greater range of distances across the cortex and better delineated primary cortical areas (see calcarine sulcus for example). (B) Variance across subjects of distance-to-connected-areas as a function of threshold. Standard deviation of distance-to- connected-areas across subjects was larger for stricter thresholds. (C) Test-retest reliability of distance-to-connected-areas as a function of threshold. Intra-class correlation coefficient was constant across thresholds. (D) Difference between geodesic and Euclidean derived maps as a function of threshold. Using either geodesic or Euclidean distances resulted in similar maps. The difference values (geodesic - Euclidean) were derived from the standardized distance metrics (ranging between 0-1 both for geodesic and Euclidean) to avoid effects of different brain size across subjects. Dispersion was largest for lenient thresholds and in areas high in distance-to-connected-areas.

### Online Resource Figure 3


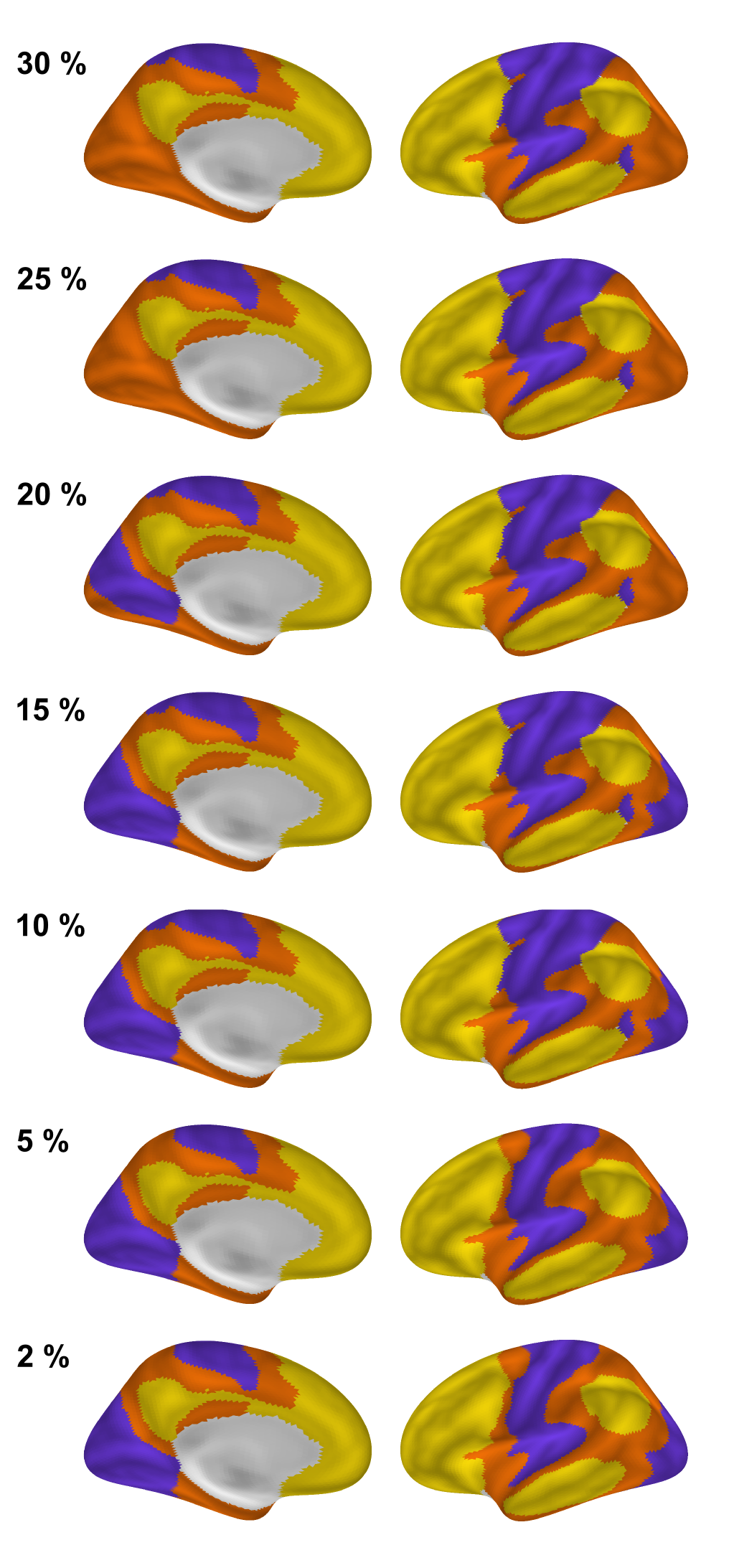


**Effect of connectivity threshold on network groupings.** To assess whether distance-to-connected-areas differentiates between different functional domains, we described intrinsic networks based on their distribution of distance-to-connected-areas. Pairwise similarity between distributions using the Jensen-Shannon divergence measure was used to cluster networks into three groups. Only at strict thresholds is the visual network grouped together with sensory-related networks.

### Online Resource Figure 4


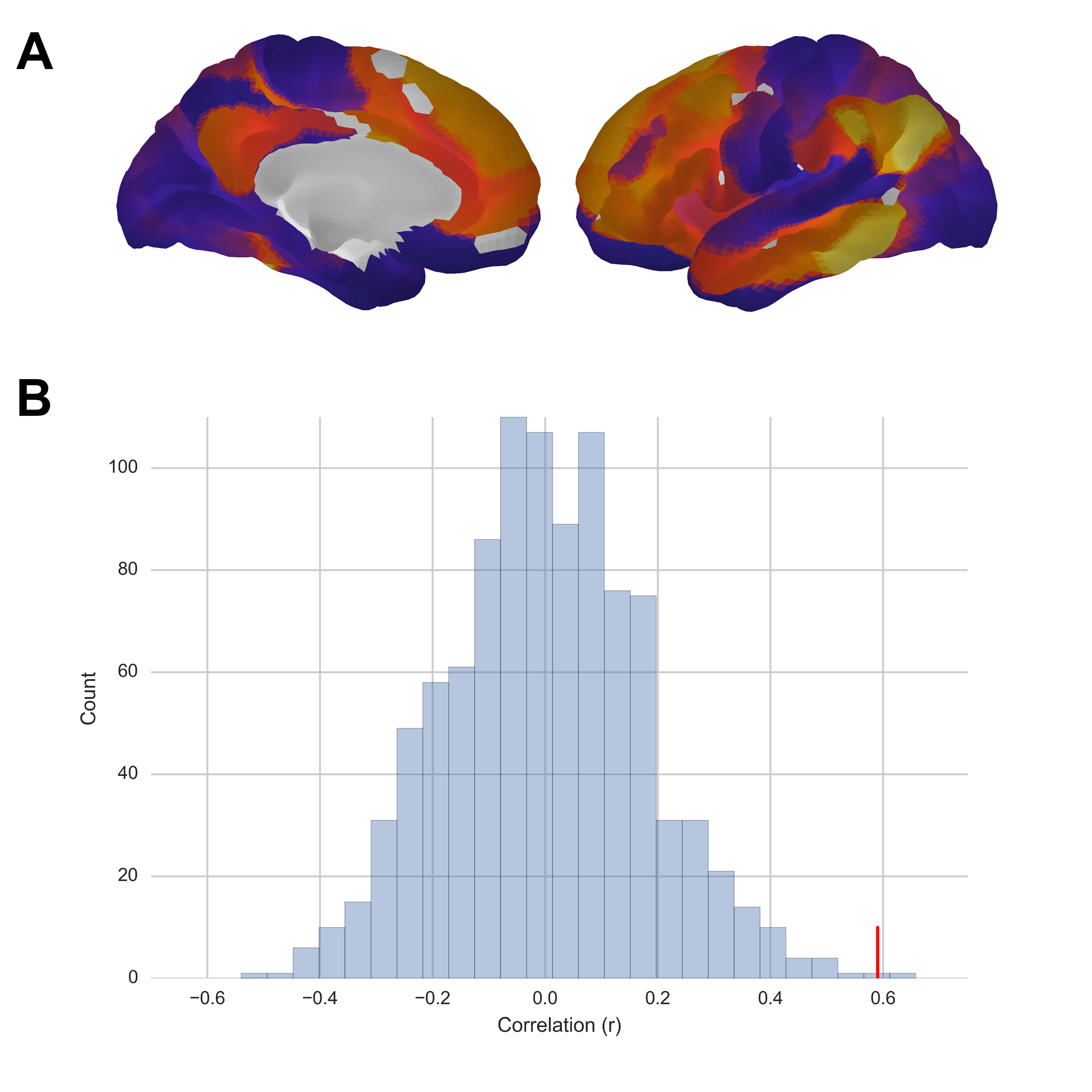


**Statistical evaluation of the relationship between distance-to-connected-areas and location of primary cortex.** (A) Functional connectivity was modelled using a binary graph with edges linking surface vertices within but not between networks. The binary connectivity model based on the original network arrangement captured the topography of distance-to-connected-areas well (spatial correlation between both maps was r = .74). Network topography was then permuted and both the distance-to-connected-areas and its relationship to the new locations of primary cortex was recalculated. (B) An empirical frequency distribution of the correlation between connectivity distance and location of primary cortex under random network topography was generated by 1000 iterations. Under random network topography, the probability of a systematic relationship as observed for the original network arrangement (r = .59, red line) was p < .002.
